# Primary Malignant Melanoma of the Oropharynx: A Rare Case of a Tonsillar Melanotic Lesion

**DOI:** 10.7759/cureus.82637

**Published:** 2025-04-20

**Authors:** Mazin J Albaldawy, Tamer Ebaied, Humaid O Al Shamsi, Ashraf Alkinain, Rahil U Faruk Abbu

**Affiliations:** 1 Department of Oncology, Al Dhannah Hospital, Abu Dhabi, ARE; 2 Department of Ear, Nose, and Throat, Al Dhannah Hospital, Abu Dhabi, ARE; 3 Department of Laboratory Services, Al Dhannah Hospital, Abu Dhabi, ARE; 4 Department of Biomedical Sciences, The University of Chicago Medicine, Chicago, USA

**Keywords:** amelanotic melanoma, histopathology and immunohistochemistry, melanocytes, oral and oropharyngeal cancer, oral malignant melanoma, tonsillar cancer

## Abstract

Oral malignant melanoma (OMM) is a rare and aggressive malignancy arising from melanocytes in the oral mucosa. We present the case of a 33-year-old Ethiopian female who arrived at the ED with hematemesis, active oral bleeding, and a sensation of suffocation. On examination, a darkly pigmented, ulcerated mass was identified arising from the left posterior tonsillar pillar. Emergency surgery was performed to control the bleeding, and subsequent histopathological analysis confirmed a diagnosis of amelanotic malignant melanoma, supported by immunohistochemical positivity for S100, HMB45, Melan-A, and P16. Advanced imaging with PET-CT and MRI demonstrated a localized tumor without evidence of lymph node involvement or distant metastasis. The patient was treated with neoadjuvant immunotherapy using pembrolizumab, followed by curative-intent radiation therapy targeting high-risk mucosal regions and bilateral cervical nodes. She tolerated the treatment well, though she experienced radiation-induced mucositis, dermatitis, and weight loss. Immunotherapy was resumed post-radiation, and the patient has since completed ten cycles without significant immune-related adverse events. The rarity of OMM and its aggressive clinical behavior underscore the need for heightened clinical suspicion for pigmented or ulcerated oral lesions. This case illustrates the diagnostic and therapeutic complexities of primary oropharyngeal melanoma, especially in the context of amelanotic presentation. It emphasizes the importance of prompt multidisciplinary evaluation, integration of advanced imaging modalities, immunohistochemical profiling, and emerging immunotherapeutic strategies to improve patient outcomes.

## Introduction

Epidemiology and demographics

Primary malignant melanoma of the oropharynx, particularly involving the tonsils, is an exceptionally rare and aggressive malignancy that originates from melanocytes. It accounts for less than 1% of all melanomas and approximately 1.6% of head and neck malignancies [1]. Oral malignant melanoma (OMM) primarily affects older individuals, with an average age at diagnosis of 56 years. The peak age of diagnosis varies by sex, occurring between 51 and 60 years in men and between 61 and 70 years in women. A notable gender disparity exists, with a male-to-female ratio of nearly 2:1 [2]. This difference becomes more pronounced with age, as men are 1.55 times more likely than women to develop melanoma by age 65 [3]. The higher incidence in men may contribute to the relatively lower average age at diagnosis observed in women.

Pathogenesis

The oropharynx, a critical component of the upper aerodigestive tract, is located posterior to the oral cavity and includes structures such as the soft palate, base of the tongue, tonsils, and the posterior pharyngeal wall. It plays essential roles in speech, swallowing, and respiration and is lined with mucosal tissue rich in melanocytes, the pigment-producing cells from which OMMs can arise. The oropharynx’s complex anatomy and deep location often contribute to diagnostic delays, as lesions in this region may not be easily visible during routine examination and can mimic more common benign or inflammatory conditions [1-3].

Unlike cutaneous melanoma, which is strongly associated with ultraviolet (UV) light exposure, OMM develops independently of UV radiation and is more commonly linked to pre-existing pigmented lesions, which may represent undiagnosed melanomas in the radial growth phase. Many of these lesions remain unrecognized until they progress into an advanced, invasive stage, highlighting the challenges of early detection [2]. The development of OMM is largely driven by genetic mutations and dysregulated signaling pathways, which contribute to uncontrolled melanocyte proliferation, resistance to apoptosis, and invasion into surrounding tissues. One of the most well-established genetic alterations in OMM is the KIT proto-oncogene, receptor tyrosine kinase mutation, which is present in up to 40% of cases. This mutation results in constitutive activation of the c-KIT receptor and downstream signaling cascades, including PI3K, JAK/STAT, and Ras-Raf-MAPK pathways, all of which play critical roles in melanocyte survival and tumor progression [4,5]. In addition to KIT mutations, polymorphisms in the melanocortin 1 receptor (MC1R) gene have been implicated in OMM pathogenesis. These genetic variations contribute to impaired DNA repair mechanisms, increased oxidative stress, and the leakage of toxic intermediates such as quinones from melanosomes, further promoting oncogenic transformation. Another key driver of tumor invasiveness is cadherin dysregulation, whereby downregulation of E-cadherin and upregulation of N-cadherin disrupt cell-cell adhesion and promote tumor spread through epithelial-mesenchymal transition (EMT) [4]. Among the most significant genetic alterations in OMM, KIT mutations are the most prevalent, with reported frequencies ranging from 11.1% to 40%. This is in stark contrast to cutaneous melanoma, where B-Raf proto-oncogene, serine/threonine kinase (BRAF) and neuroblastoma RAS viral oncogene homolog (NRAS) mutations predominate [4,5]. Recently, novel FMNL2 mutations have been identified in 22% of OMM cases, although their precise role in tumor progression remains unclear [5]. These discoveries underscore the therapeutic potential of targeted approaches, particularly in KIT-mutated OMM, which may respond to tyrosine kinase inhibitors (TKIs) such as imatinib or dasatinib. While high mitotic rate and perineural invasion are well-established markers of aggressive behavior in many cancers, these features have not yet been comprehensively evaluated in OMM [4-6].

Clinical presentation and diagnosis

OMM exhibits a distinct predilection for specific sites within the oral cavity, with the hard palate being the most frequently affected location. The distribution of these melanomas is well documented, with the hard palate accounting for 34-40% of cases, followed by the maxillary gingiva as the second most common site. Collectively, the palate and maxillary gingiva contribute to approximately 80% of oral melanoma cases [2]. Clinically, OMM typically presents as a pigmented swelling or mass, with coloration ranging from dark brown to blue-black, though variations in gray, purple, and red may also be observed. However, amelanotic melanomas, which constitute 5-35% of cases, can appear as white, mucosa-colored, or red masses, often leading to misdiagnosis as benign lesions or inflammatory conditions, further complicating early recognition [2]. As OMM progresses, patients may experience ulceration, spontaneous bleeding, tooth loss, and difficulties with dental prostheses, significantly impairing oral function and reducing quality of life [7]. In later stages, regional lymphadenopathy often indicates metastatic spread and portends a poorer prognosis [2]. OMM can be classified into in situ, invasive, and mixed patterns, each influencing disease progression and treatment strategies. Staging is typically categorized into Stage I (localized disease), Stage II (lymph node metastasis), and Stage III (distant metastases), with each stage carrying important prognostic implications. OMM displays considerable morphological diversity, which further complicates diagnosis. Histologically, it can be classified into five subtypes: pigmented nodular, nonpigmented nodular (amelanotic melanoma), pigmented macular, pigmented mixed, and nonpigmented mixed [8]. Tumor cells may exhibit epithelioid morphology (50%), spindle-shaped morphology (50%), or a combination of both, with 37.5% of cases demonstrating round, blue, or clear cells [6]. The degree of pigmentation also varies significantly: while 53% of cases show melanin deposition, amelanotic variants are frequently misdiagnosed due to their resemblance to benign lesions or other mucosal malignancies, leading to delayed diagnosis and treatment [4,6]. Tonsillar melanoma, a particularly aggressive and rare form of mucosal melanoma, is typically characterized by rapid progression and high metastatic potential. These tumors often appear as bluish-black masses, though secondary metastatic cases from cutaneous melanoma have also been reported [9]. Histopathological analysis plays a central role in distinguishing OMM from other oral neoplasms. Immunohistochemical markers such as HMB45, S100, and Melan-A are widely used to confirm melanocytic origin, aiding in differentiation from squamous cell carcinoma, lymphoma, or other mucosal malignancies [9]. Molecular profiling for mutations in KIT, NRAS, and BRAF genes is also recommended, as these genetic alterations may guide the selection of targeted therapies [4,5]. Given OMM’s high metastatic potential, advanced imaging techniques are critical for tumor staging and treatment planning. In early-stage disease, imaging helps define tumor margins and detect subclinical spread, while in advanced cases, it is useful for assessing metastatic burden and guiding palliative or curative treatment approaches. Recommended modalities include MRI for evaluating tumor depth, soft tissue invasion, and perineural spread; contrast-enhanced CT for assessing bony involvement and regional lymphadenopathy; and PET-CT for detecting distant metastases and evaluating treatment response [8].

Treatment strategies

The primary approach to treating OMM involves surgical resection, which remains the cornerstone of management. Wide local excision with tumor-free margins is critical to achieving disease control and improving survival outcomes [2]. In cases where lymph node involvement is suspected, a neck dissection may be performed to reduce the risk of regional metastatic spread [10]. Immunotherapy has emerged as a promising adjunctive treatment. In one documented case of recurrent OMM, pembrolizumab combined with topical imiquimod achieved complete histopathological remission [10]. Other immunotherapeutic approaches, including interleukin-2 and immune checkpoint inhibitors, have also been explored to reduce recurrence risk and enhance immune-mediated tumor suppression [11]. For tumors harboring specific genetic mutations, targeted therapy provides an effective alternative. Patients with BRAF or KIT mutations may benefit from BRAF inhibitors, which selectively inhibit mutated signaling pathways, thereby slowing tumor progression and improving clinical outcomes [12].

Prognosis and follow-up

The prognosis for OMM remains poor, with five-year survival rates ranging from 20% to 35%. High recurrence rates necessitate lifelong follow-up. In a documented case, a patient remained in remission for four years before developing melanoma in situ, requiring additional treatment [10]. Compared to cutaneous melanomas, mucosal melanomas, including OMM, exhibit a more aggressive disease course, reflected in their lower five-year survival rates [13]. This increased aggressiveness is partially attributed to the tumor microenvironment, which is characterized by low immunogenicity, rendering OMM less responsive to immunotherapies that have transformed the treatment of cutaneous melanoma [10]. Additionally, the anatomical location of oral tumors complicates treatment, as complete surgical excision may be difficult without causing significant functional or aesthetic impairment [14]. Another factor contributing to the poor prognosis of OMM is its limited response to both targeted therapies and immunotherapies. Although immune checkpoint inhibitors have revolutionized cutaneous melanoma management, their effectiveness in mucosal melanomas remains limited. This is thought to result from the lower mutation burden in mucosal tumors, which reduces the likelihood of a strong immune response. Furthermore, mucosal melanomas exhibit higher frequencies of KIT mutations rather than BRAF mutations, making standard BRAF-targeted therapies less effective in these cases [10].

Risk factors

A major concern with OMM is its silent progression, as these lesions often remain asymptomatic until the disease has significantly advanced. Since most individuals do not routinely examine their oral cavity, melanomas may go unnoticed until symptoms such as swelling, tooth mobility, or bleeding prompt medical attention [2]. The rarity and specific site predilection of oral mucosal melanomas present significant diagnostic challenges. Due to their asymptomatic nature, these malignancies often develop silently, with few noticeable symptoms until substantial disease progression has occurred. Additionally, delayed detection is common, as most individuals do not routinely inspect their oral cavity, allowing lesions to remain undetected until symptoms become prominent [2,15]. This delay significantly impacts treatment outcomes, as oral melanomas are often diagnosed at an advanced stage, when therapeutic options are less effective. Another complicating factor in early detection is misdiagnosis, particularly in amelanotic melanomas, which lack pigmentation and may be mistaken for benign lesions or other malignancies such as squamous cell carcinoma [16]. Given that amelanotic variants account for 5-35% of OMM cases, this diagnostic ambiguity frequently leads to delays in appropriate treatment and contributes to poorer prognoses [8].

This case report highlights the critical importance of early detection and comprehensive management in primary malignant melanoma of the oropharynx. Given its rarity and aggressive clinical behavior, clinicians must maintain a high index of suspicion for pigmented or atypical lesions in the oral cavity, particularly in high-risk anatomical sites such as the palate and maxillary gingiva. Timely diagnostic evaluation, including biopsy and advanced imaging, paired with multidisciplinary care, is essential for improving patient outcomes. OMM presents unique challenges due to its silent progression, site-specific limitations, high rates of misdiagnosis, and limited effective treatment options, all of which collectively contribute to its poor prognosis. Early recognition, improved screening strategies, and optimized therapeutic approaches are therefore crucial to enhancing long-term survival. Given the tumor’s aggressive nature and high recurrence potential, coordinated care involving surgeons, oncologists, and radiation therapists is vital. While recent advances in molecular and immunotherapeutic strategies offer hope, continued research is needed to expand treatment efficacy and reduce recurrence. In particular, there is an urgent need for the identification of prognostic biomarkers that can support risk stratification and guide personalized therapeutic decision-making, especially in cases characterized by late detection, early lymphatic dissemination, and resistance to systemic therapies.

## Case presentation

Initial consultation and surgery

**Figure 1:**
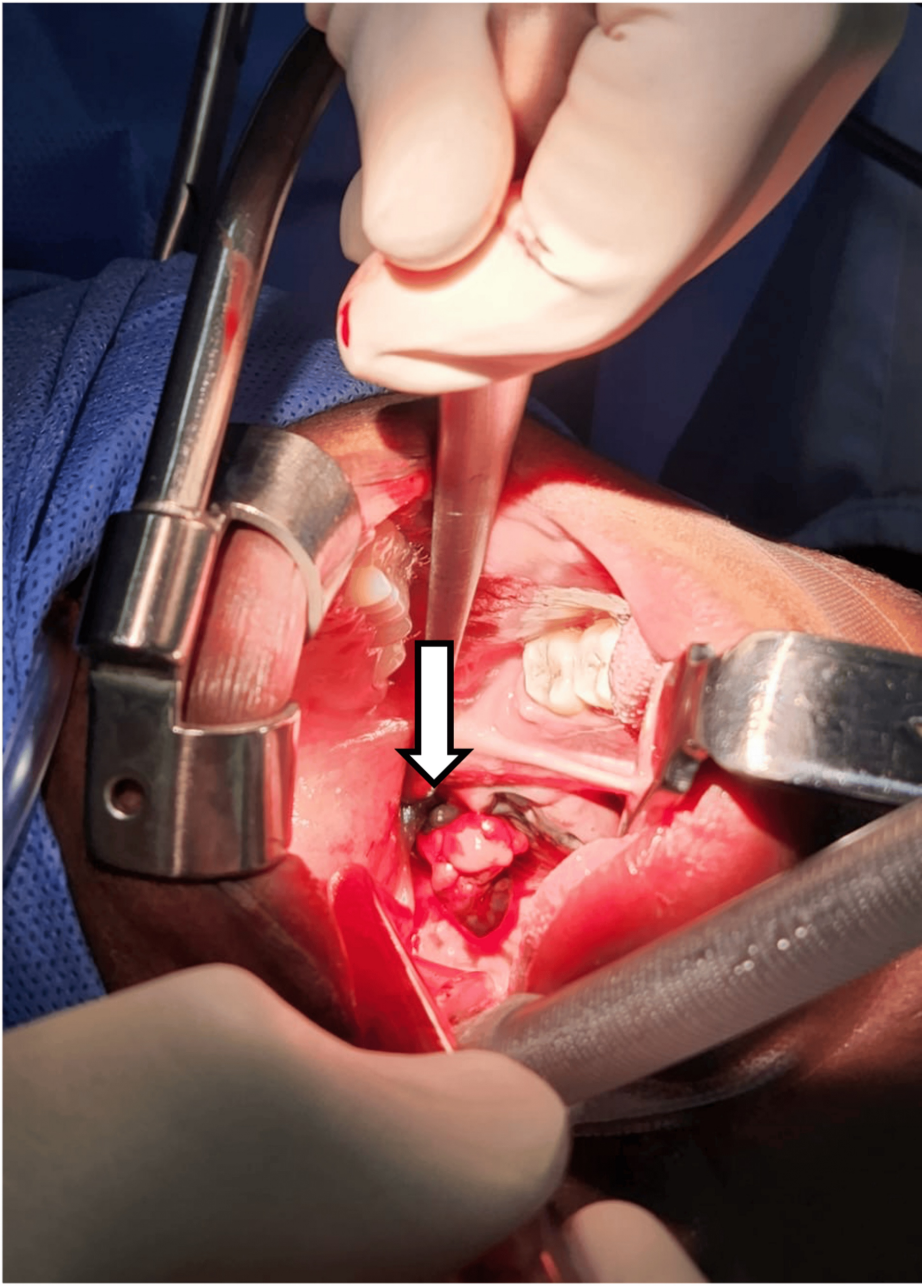
Intraoperative view of malignant melanoma arising from the left posterior tonsillar pillar. The image highlights a blackish lesion with surface ulceration, consistent with primary malignant melanoma of the oropharynx. Surgical instruments and retractors are used to maintain a clear operative field for tumor resection and control of active bleeding. This image is published with the patient’s written informed consent, in accordance with institutional and ethical guidelines.

A 33-year-old Ethiopian female, single, nulliparous, and a non-smoker with no history of alcohol use or family history of cancer, presented to the ED with complaints of hematemesis, active oral bleeding, and a sensation of suffocation. She reported no known allergies, no significant past medical history, and no prior surgeries. The patient first noticed black spots on her tonsils two months earlier, accompanied by throat pain and progressively worsening dysphagia, which prompted her to seek medical attention. Evaluation by an otolaryngologist revealed a lesion hanging behind the left tonsil, arising from the left posterior tonsillar pillar, with blackish discoloration of the oropharyngeal walls bilaterally. On examination in the ED, the same lesion was noted with surface ulceration, accounting for the oral bleeding. The patient’s overall health status was otherwise unremarkable, and she was not taking any medications. Emergency surgery was performed to control the bleeding (Figure 1), and the excised specimen was sent for pathological evaluation.

Imaging

Following the emergency surgical intervention, the patient underwent further diagnostic workup, including MRI and PET-CT, to assess the extent of the lesion and rule out metastasis. These modalities were employed to evaluate the local extent of the primary lesion, assess involvement of adjacent anatomical structures, and exclude regional or distant metastatic disease.

PET-CT

**Figure 2:**
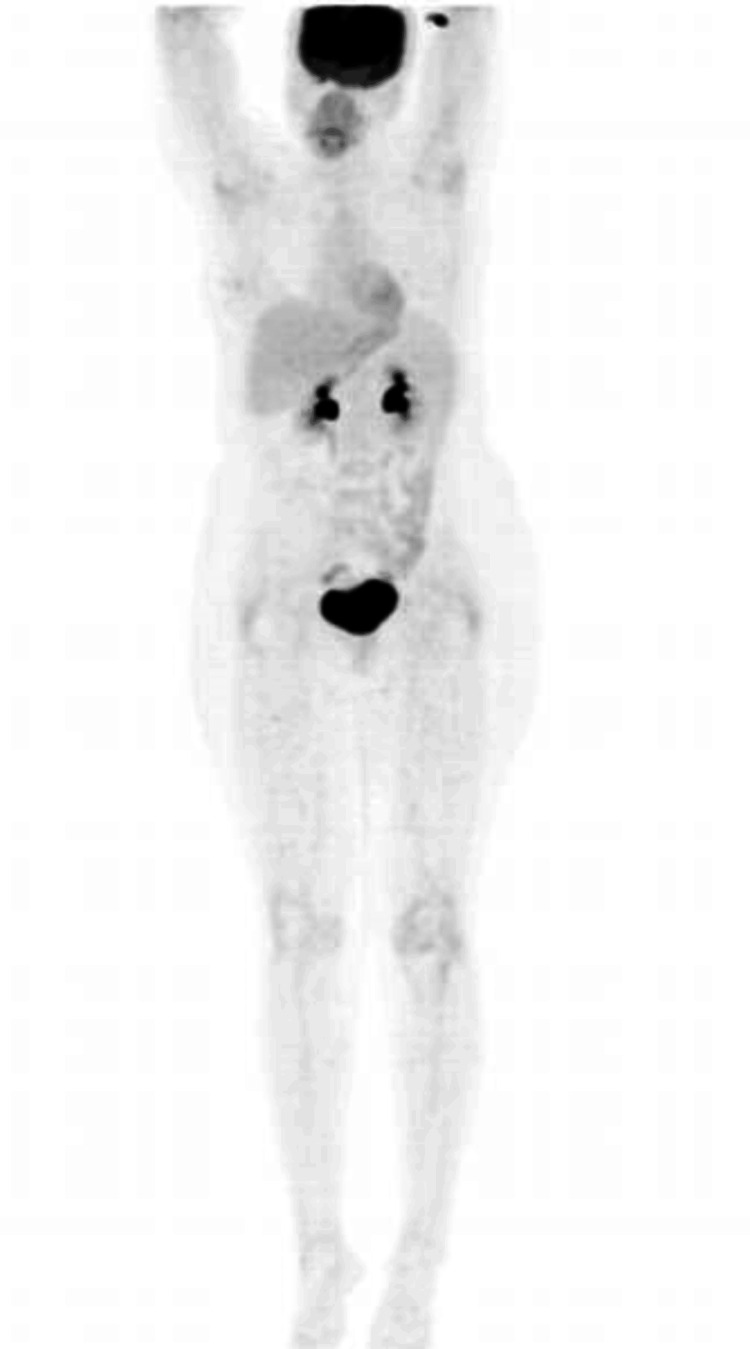
Whole-body PET-CT scan image. The scan reveals no scintigraphic evidence of FDG-avid focal lesions in the skin or subcutaneous tissues, indicating the absence of metabolically active disease at the time of imaging. FDG: Fluorodeoxyglucose.

The PET-CT scan (Figure 2) revealed no scintigraphic evidence of fluorodeoxyglucose (FDG)-avid focal lesions in the skin or subcutaneous tissues on whole-body survey. There was also no evidence of FDG-avid lymphadenopathy or distant organ involvement. These findings suggest the absence of metabolically active disease in the skin, subcutaneous tissues, lymph nodes, or visceral organs, indicating no detectable metastatic or systemic spread at the time of imaging.

While OMMs are typically FDG-avid due to their high metabolic activity, this case was atypical. Neither the primary lesion nor any metastatic sites demonstrated FDG uptake. Several factors may account for this discrepancy. First, the tumor was predominantly amelanotic, a phenotype known to exhibit lower metabolic activity, which may result in reduced FDG uptake. Second, the primary lesion’s small size and superficial mucosal location likely placed it below the spatial resolution threshold of PET-CT, particularly for detecting lesions under 10 mm. Finally, the PET-CT was performed after surgical excision of the primary lesion, which may have contributed to the absence of FDG activity at the primary site.

MRI

**Figure 3:**
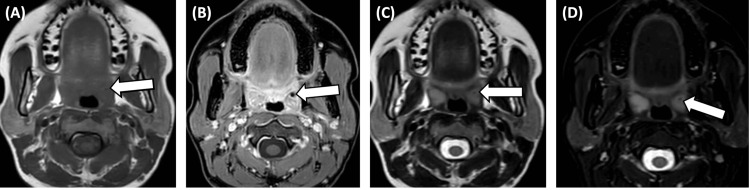
MRI evaluation of the left palatine tonsil. (A) Axial T1-weighted pre-contrast image demonstrating a focal hypointensity in the left palatine tonsil, likely representing post-excision biopsy changes, with no evidence of residual or recurrent mass. The right palatine tonsil and posterior pharyngeal wall appear normal. (B) Axial T2-weighted pre-contrast image showing the same focal hypointensity in the left palatine tonsil without additional abnormalities. (C) Axial T2-weighted image providing clearer delineation of the hypointense area, confirming its localized nature and absence of contrast enhancement. (D) Axial short tau inversion recovery (STIR) image highlighting the hypointense region with fat signal suppression, consistent with post-biopsy changes. In all panels, the cranial end of the image is oriented toward the top, and the caudal end toward the bottom. Left and right correspond to the patient’s left and right sides, respectively, as per standard radiologic convention.

MRI demonstrated a midline pharyngeal mucosal lesion measuring 1.5 × 1.5 cm. The lesion showed heterogeneous high signal intensity on T2-weighted sequences (Figure 3B) and heterogeneous enhancement on post-contrast imaging. Tiny T2 hyperintensities were noted adjacent to the right fossa of Rosenmüller, however, there was no evidence of disease extension beyond the submucosa. The mastoid air cells were clear, and the prominent bilateral palatine tonsils showed no focal lesions (Figures 3A-3B). A focal signal void was identified in the left palatine tonsil across all imaging sequences, most likely representing post-biopsy changes (Figures 3A-3D). No cervical lymphadenopathy or enlarged lymph nodes were detected based on size criteria. The thyroid, parotid, and submandibular glands appeared normal in morphology and signal intensity. The aerodigestive tract and major neck vessels were unremarkable. Vertebral alignment and height were well preserved, with normal bone marrow signal intensity (Figure 3C). The visualized brain and orbits also showed no abnormalities. These findings are consistent with a midline pharyngeal mucosal lesion and tiny T2 hyperintensities near the right fossa of Rosenmüller, without submucosal extension or evidence of regional or distant metastatic spread.

Histopathology

**Figure 4:**
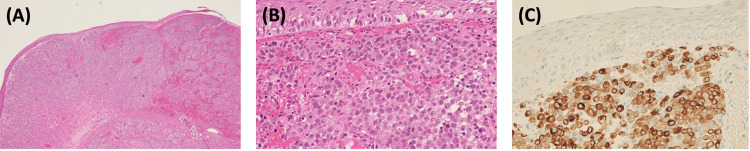
Microscopic imaging of primary malignant melanoma of the oropharynx using hematoxylin and eosin and immunohistochemical staining. (A) H&E staining at low magnification (4×) demonstrates a polypoid fragment lined by ulcerated stratified squamous epithelium. The underlying stroma is infiltrated by malignant cells arranged in nests with variable pigmentation. (B) H&E staining at high magnification (40×) shows extensive infiltration of tumor nests within the subepithelial stroma. Tumor cells exhibit marked pleomorphism, hyperchromatic nuclei, prominent nucleoli, and a high mitotic index, consistent with an aggressive melanoma phenotype. (C) Immunohistochemical staining for Melan A at 40× magnification shows strong cytoplasmic positivity in tumor cells, confirming melanocytic origin. Brown-stained regions highlight Melan A expression, a marker specific to melanocytes.

Microscopic analysis revealed a polypoid fragment lined by extensively ulcerated stratified squamous epithelium. The subepithelial stroma exhibited dense infiltration by a malignant tumor arranged in nests and composed of epithelioid cells with vesicular nuclei and prominent nucleoli (Figure 4B). The mitotic index was 1 per 10 high-power fields (HPFs). While focal pigmentation was observed, the tumor was predominantly amelanotic. Immunohistochemistry supported the diagnosis, with tumor cells staining positively for S100, HMB45, Melan A, and P16 (Figure 4C), and negatively for Desmin, CD99, AE1/3, Cam5.2, CK7, Chromogranin, Calretinin, CD56, CD45, P63, and Synaptophysin. In situ hybridization for Epstein-Barr virus RNA (EBV-RNA) was negative. The Ki-67 proliferation index was markedly elevated, indicating high proliferative activity. The depth of invasion could not be precisely measured due to poor orientation; however, all tissue fragments were involved, with the largest measuring 17 mm and entirely infiltrated by tumor. Focal pagetoid spread was noted in viable mucosa, but assessment was limited due to extensive ulceration. No features of regression were identified. The presence of bizarre, pleomorphic cells with prominent nucleoli is clearly demonstrated in Figure 4C. Together, the morphological characteristics (Figures 4A-4B) and immunohistochemical profile confirmed the diagnosis of nodular malignant melanoma arising from the visceral oropharynx.

Follow-up

The patient was initiated on a neoadjuvant immunotherapy regimen consisting of pembrolizumab (Keytruda) at a dose of 400 mg administered intravenously every six weeks. This was later adjusted to 200 mg every three weeks. She completed six cycles of immunotherapy with a favorable clinical response; however, she was ultimately deemed not a suitable candidate for surgical resection. Following multidisciplinary team discussion, the patient underwent curative-intent radiation therapy using volumetric modulated arc therapy (VMAT) with simultaneous integrated boost (SIB) and daily image-guided radiotherapy (IGRT). High-risk mucosal areas, including the oropharynx, nasopharynx, and hypopharynx, were treated to a total dose of 66 Gy in 33 fractions. Low-risk regions, including bilateral cervical and retropharyngeal lymph nodes, received 56 Gy in 33 fractions. The course was delivered over 46 days with only one day of treatment interruption. The patient tolerated radiation well and completed therapy without major disruptions.

Toward the end of treatment, she developed acute side effects, including Grade 1 fatigue, Grade 2-3 oral and pharyngeal mucositis, and Grade 2 radiation dermatitis. These were managed symptomatically using Aquaphor cream for skin care and salt-soda gargles every 4-6 hours. Severe mucositis led to approximately 7 kg of weight loss due to reduced oral intake; although a feeding tube was recommended, the patient declined placement. She was counseled that symptoms might worsen transiently post-radiation and was provided with detailed oral and dermatologic care instructions to support recovery. Upon resolution of acute toxicities, pembrolizumab was resumed. She has since completed 10 of the planned 17 immunotherapy cycles without significant immune-related adverse events. Thyroid function and adrenocorticotropic hormone (ACTH) levels are monitored at each cycle to assess for endocrine side effects, with no abnormalities detected to date. The patient continues under close surveillance to monitor treatment response, manage any late-onset toxicities, and support nutritional and functional recovery. Follow-up imaging, including repeat MRI and PET-CT, is scheduled to reassess disease status and guide further management. She remains under the joint care of her referring physicians at Al Dhannah Hospital and the radiation oncology team for ongoing monitoring of disease progression and late radiation effects.

## Discussion

OMM is a rare and aggressive malignancy arising from melanocytes in the oral mucosa, with significant challenges in diagnosis, treatment, and management due to its variable clinical presentations and rapid progression. This discussion builds upon the case of a 33-year-old Ethiopian female who presented with hematemesis, active oral bleeding, and a sensation of suffocation, and was ultimately diagnosed with OMM originating from the left posterior tonsillar pillar.

The clinical presentation of OMM is often insidious, as demonstrated in this case, where the patient initially noticed black spots and throat discomfort two months prior to diagnosis. Such delays in seeking medical attention are common, as OMM frequently mimics benign or inflammatory conditions. This observation is consistent with findings by Kukde MM et al. [17], who reported similar diagnostic delays, particularly in amelanotic variants. Aloua R et al. [18] also emphasized the diagnostic challenges posed by the non-specific appearance of OMM lesions. Likewise, Manigandan T et al. [19] described a case that was initially misdiagnosed as a benign lesion, reinforcing the importance of maintaining a high index of suspicion for pigmented or ulcerated oral findings. In the present case, the lesion’s location on the left posterior tonsillar pillar, along with its ulcerated surface, contributed to active bleeding and necessitated emergency surgical intervention. The presence of satellite foci and the combination of smooth and ulcerated regions further complicated the clinical assessment.

Given the silent yet rapid progression characteristic of oral mucosal melanoma, a clear understanding of the patient's clinical course is critical. The sequential unfolding of symptoms, diagnostic interventions, and therapeutic milestones emphasizes the urgency of early recognition, prompt surgical decision-making, and coordinated multidisciplinary care in managing such aggressive pathologies (Table 1).

**Table 1:** Clinical timeline of diagnosis, treatment, and follow-up. This table summarizes the month-by-month progression of clinical symptoms, diagnostic interventions, and therapeutic management in the presented case of OMM. It highlights the rapid disease progression and underscores the necessity of prompt, multidisciplinary intervention to achieve optimal patient outcomes. OMM: Oral malignant melanoma; VMAT: Volumetric modulated arc therapy; SIB: Simultaneous integrated boost.

Month	Clinical Event
December 2024	Patient noticed black spots on tonsils with throat pain and dysphagia. However, no medical evaluation was sought by the patient.
Late January 2025	Symptoms worsened, with new onset of hematemesis and a sensation of suffocation.
Early February 2025	Presented to the emergency department at Al Dhannah Hospital with a pigmented lesion identified on the left posterior tonsillar pillar. On the same day, emergency surgical excision was performed to control active bleeding.
Mid-February 2025	PET-CT, MRI, and histopathological analysis confirmed amelanotic malignant melanoma.
Late February 2025	Initiation of neoadjuvant immunotherapy with pembrolizumab.
Late March 2025	Underwent curative-intent radiation therapy (VMAT with SIB) over a 46-day course.
April 2025 (Current)	Ongoing immunotherapy. Patient remains under regular multidisciplinary follow-up with no major immune-related toxicities.

Imaging modalities played a critical role in the diagnosis and staging of OMM in this case. MRI was instrumental in delineating tumor margins, assessing soft tissue involvement, and guiding surgical planning. Its superior soft tissue contrast enabled precise visualization of lesion extent, consistent with findings by Kukde MM et al. [17], who emphasized MRI’s value in differentiating benign from malignant lesions and detecting lymphadenopathy. PET-CT provided additional insights by identifying FDG-avid lesions indicative of metabolically active disease. This was essential for ruling out distant metastases and evaluating systemic tumor burden. Aloua R et al. [18] similarly reported PET-CT’s utility in detecting metastatic spread to common sites such as the lungs and in monitoring treatment response. Although PET imaging was not included in the case reported by Manigandan T et al. [19], the authors acknowledged its complementary role to CT in functional disease assessment. The comparison between MRI and PET-CT demonstrates their complementary roles in OMM management: MRI excels in evaluating local disease extent and surgical feasibility, while PET-CT is superior in identifying systemic involvement and assessing therapeutic efficacy. Together, these modalities enable comprehensive disease evaluation and enhance diagnostic accuracy.

Histopathological analysis of the excised specimen in the present case revealed infiltration by malignant melanocytes, predominantly amelanotic, consistent with findings reported by Kukde MM et al. [17] and Aloua R et al. [18]. Immunohistochemistry confirmed the diagnosis, with tumor cells expressing S100, HMB45, and Melan A, markers essential for differentiating OMM from other oral neoplasms. Manigandan T et al. [19] likewise emphasized the importance of immunohistochemical staining in establishing a definitive diagnosis, particularly in cases with atypical histological features.

As outlined previously, the patient in this case underwent emergency surgery to control bleeding, followed by further diagnostic evaluation and consideration for adjuvant therapy. Modern treatment strategies, such as neoadjuvant immunotherapy with pembrolizumab, reflect evolving approaches to managing advanced melanoma. Kukde MM et al. [17] and Aloua R et al. [18] also highlighted the role of immunotherapy as a promising option for OMM, particularly in cases with high metastatic potential. In contrast, Manigandan T et al. [19] reported a more traditional approach involving surgical excision followed by adjuvant therapy, illustrating variability in treatment strategies based on individual patient factors and tumor characteristics.

Prognosis remains poor for OMM due to its aggressive nature and high rates of recurrence and metastasis, as evidenced by the elevated Ki-67 proliferation index observed in this case, indicating heightened tumor activity. After completing multiple cycles of pembrolizumab with a favorable clinical response, the patient underwent curative-intent radiation therapy using advanced techniques such as VMAT with SIB. Despite completing radiation with minimal interruptions, she developed acute side effects including fatigue, mucositis, and dermatitis, all of which were managed symptomatically. Following recovery from radiation-induced toxicities, immunotherapy was resumed as part of a planned 17-cycle regimen. This proactive, multidisciplinary approach, incorporating advanced imaging, structured follow-up, and nutritional support, remains essential for assessing treatment response, monitoring recurrence, and managing long-term effects. In contrast, Manigandan T et al. [19] described a case in which the patient received palliative external beam radiotherapy and experienced poor outcomes due to limited treatment response and less comprehensive follow-up. Similarly, Kukde MM et al. [17] reported substantial disease progression despite treatment, underscoring the aggressive course of OMM and the difficulty in achieving sustained remission. In that instance, follow-up focused primarily on survival rather than a structured system for monitoring treatment effects. These comparisons emphasize the importance of a vigilant, multidisciplinary strategy to optimize care and improve outcomes for patients with OMM.

In summary, this case illustrates how a convergence of atypical clinical features, namely amelanosis, posterior tonsillar pillar involvement, and a prominently ulcerative lesion, enabled distinction from more common oropharyngeal pathologies. The lesion’s amelanotic nature masked the classical pigmentation typically associated with melanoma, increasing the likelihood of misclassification as a benign inflammatory process. Its origin from the posterior tonsillar pillar, an anatomically concealed and infrequently visualized region, further contributed to diagnostic delay. Additionally, hallmark features of oral mucosal melanoma, including aggressive local invasion, high mitotic activity, and early regional or distant spread, were mirrored in this case by active bleeding, satellite foci, and a mixed smooth-ulcerated morphology. These clinical signs, when interpreted alongside corroborative histopathological and immunohistochemical findings outlined above, were critical in establishing a timely and accurate diagnosis. Together, they underscore the need for heightened clinical vigilance, especially in evaluating rapidly evolving oropharyngeal lesions with atypical morphology. Ultimately, this case reinforces the importance of integrating clinical, radiological, and pathological data in the early identification and multidisciplinary management of rare but aggressive malignancies such as OMM.

## Conclusions

A thorough and multidisciplinary approach is essential for the diagnosis and management of OMM, particularly when clinical presentations are atypical. The rarity and aggressive nature of OMM demand a high index of suspicion for pigmented or ulcerated oral lesions, as delays in diagnosis can lead to significantly worse outcomes. In this case, advanced imaging modalities, including MRI for local disease assessment and PET-CT for systemic evaluation, provided a comprehensive understanding of the tumor’s extent and metabolic activity. Histopathological and immunohistochemical analyses, utilizing markers such as S100, HMB45, and Melan A, were crucial for definitive diagnostic confirmation and differentiation from other neoplasms. The integration of modern treatment strategies, including neoadjuvant immunotherapy, demonstrates the evolving landscape of melanoma management and highlights the potential for improved outcomes through personalized care. However, the aggressive nature of OMM, combined with its poor prognosis and high risk of recurrence and metastasis, makes it challenging to achieve favorable outcomes. This case underscores the importance of vigilant follow-up and the necessity of a collaborative, multidisciplinary team to optimize care. By contributing to the broader understanding of OMM, it reinforces the critical need to integrate clinical assessment, advanced imaging, and histopathological findings to ensure timely and effective diagnosis and treatment.
